# The Contribution of Network Organization and Integration to the Development of Cognitive Control

**DOI:** 10.1371/journal.pbio.1002328

**Published:** 2015-12-29

**Authors:** Scott Marek, Kai Hwang, William Foran, Michael N. Hallquist, Beatriz Luna

**Affiliations:** 1 Center for Neuroscience, University of Pittsburgh, Pittsburgh, Pennsylvania, United States of America; 2 Center for the Neural Basis of Cognition, Carnegie Mellon University, Pittsburgh, Pennsylvania, United States of America; 3 Helen Wills Neuroscience Institute, University of California Berkeley, Berkeley, California, United States of America; 4 Department of Psychiatry, University of Pittsburgh, Pittsburgh, Pennsylvania, United States of America; 5 Department of Psychology, The Pennsylvania State University, State College, Pennsylvania, United States of America; 6 Department of Psychology, University of Pittsburgh, Pittsburgh, Pennsylvania, United States of America; University of Oregon, UNITED STATES

## Abstract

Cognitive control, which continues to mature throughout adolescence, is supported by the ability for well-defined organized brain networks to flexibly integrate information. However, the development of intrinsic brain network organization and its relationship to observed improvements in cognitive control are not well understood. In the present study, we used resting state functional magnetic resonance imaging (RS-fMRI), graph theory, the antisaccade task, and rigorous head motion control to characterize and relate developmental changes in network organization, connectivity strength, and integration to inhibitory control development. Subjects were 192 10–26-y-olds who were imaged during 5 min of rest. In contrast to initial studies, our results indicate that network organization is stable throughout adolescence. However, cross-network integration, predominantly of the cingulo-opercular/salience network, increased with age. Importantly, this increased integration of the cingulo-opercular/salience network significantly moderated the robust effect of age on the latency to initiate a correct inhibitory control response. These results provide compelling evidence that the transition to adult-level inhibitory control is dependent upon the refinement and strengthening of integration between specialized networks. Our findings support a novel, two-stage model of neural development, in which networks stabilize prior to adolescence and subsequently increase their integration to support the cross-domain incorporation of information processing critical for mature cognitive control.

## Abstract 2

This study reveals that although the organization of functional brain networks remains stable during adolescence, between-network integration continues to increase, underlying maturation in cognitive control.

## Author Summary

Adolescence is a unique period of brain development, with major changes occurring across the brain at many different levels of brain functioning. At the macroscopic level, the brain is composed of individual regions that collaborate in networks to perform diverse cognitive functions. Some networks of brain regions perform lower-level sensorimotor processing, while other networks orchestrate more complex functions, such as cognitive control. The affiliation of each region to a network is referred to as network organization. Brain regions not only can communicate with other regions belonging to their own network but also with regions in other networks. Brain regions that communicate with regions belonging to other networks display a high level of *integration* since they link their network with another network. We found that during adolescence, network *organization* does not change. However, *integration* continues to increase, underscoring the notion that brain function becomes more distributed and collaborative during this unique period of development. Furthermore, this increased network *integration* underlies improvements in cognitive control. Thus, we provide a network-based account for improvements in cognitive functioning during adolescence.

## Introduction

Cognitive control refers to the ability to execute voluntary, goal-directed behavior [1–3]. It requires flexible and adaptive coordination of core executive systems that are supported by integration among widely distributed, specialized brain circuitries [4]. The core components of cognitive control are available early in development [5]. However, in adolescence, cognitive control abilities become significantly more reliable and flexible, as response accuracy and speed stabilize in adulthood [6]. These developmental gains in information processing occur in parallel with brain maturational events, including synaptic pruning [7] and myelination [8], which predominantly enhance collaboration among brain systems [9]. The nature of the interaction between brain network maturation and cognitive development during adolescence is not well understood [10], limiting our ability to understand the neural basis of psychopathologies that emerge at this time, many of which are characterized by deficits in cognitive control [11].

Characterizing functional brain network interactions during the resting state (i.e., while the subject is not engaged in any particular task) has become a valuable emerging approach for investigating the brain basis of cognitive development. Studies using this approach have revealed roles for these networks in supporting cognitive control [4,12]. Approximately 20 functional networks have been identified in the functional connectome [13], including sensory networks, such as the somatomotor (SM) and visual networks; cognitive networks, such as the fronto-parietal (FP) and cingulo-opercular/salience (CO/Salience) networks; and a task-negative default mode (DM) network [14]. Each functional network operates as a module within the full connectome. Networks are demarcated by dense internal connectivity [15,16], defining a foundational organization for the functional brain. Thus, network *organization* refers to the network affiliation of each region of the connectome. Initial studies characterizing age-related changes in functional network organization suggested that the organization of these networks continued to change into adulthood [17], such that development proceeded from short-distance anatomical networks in infancy and childhood, to long-range, widely distributed networks in adulthood [17–20]. However, age-related differences in head motion artifacts may have confounded the connectivity distance findings [21–23]. Advances in data processing methods [21–23] and recent findings suggest that foundational aspects of functional network *organization* are established early in development, while processes related to network *integration* continue to mature into adulthood [24]. Network *integration* refers to the level of functional coupling between networks, measured by participation coefficient (PC), a graph theoretical construct [25]. PC is a particularly useful construct to measure network integration, given its sensitivity to between-network connectivity, while maintaining robustness to the total number of connections (degree). Degree-based measures of integration have been shown to be dependent on the size (number of nodes) of a network and therefore can skew results towards a greater number of hubs within larger networks, such as the default mode network [26]. PC is normalized by the degree of the node. As a result, increases in PC are driven mainly by increases in the number of between-network connections.

Properties of network organization and integration could parallel cognitive development, which is characterized by enhanced adaptive and flexible integration of mature core control components [1]. Thus, in the present study, we sought to identify whether age-related changes in functional networks are determined by changes in network *organization* and/or network *integration* and whether these changes are related to developmental improvements in cognitive control. We applied graph theory [27,28] to a rich developmental resting-state functional magnetic resonance imaging (RS-fMRI) dataset obtained in 10–26-y-olds who also performed the antisaccade task. In this inhibitory control paradigm, subjects fixate a central target on a computer screen. A stimulus is then presented at an unpredictable horizontal location. Subjects are instructed to refrain from making a saccadic eye movement towards the stimulus (i.e., inhibitory response) and instead make a voluntary saccadic eye movement to the mirrored opposite location on the horizontal meridian.

Given that core cognitive components are on line by childhood and that the ability to adaptively and flexibly engage these components improves into adulthood [29–33], we hypothesized that network *organization*, which supports component processes, would not change with age, but that network connectivity *strength* and *integration*, which both support interaction between components, would increase with age. In turn, we hypothesized increased control network integration would predict age-related improvements in cognitive control, as measured by the antisaccade task.

## Results

### Development of Functional Network Organization

**Fig 1:**
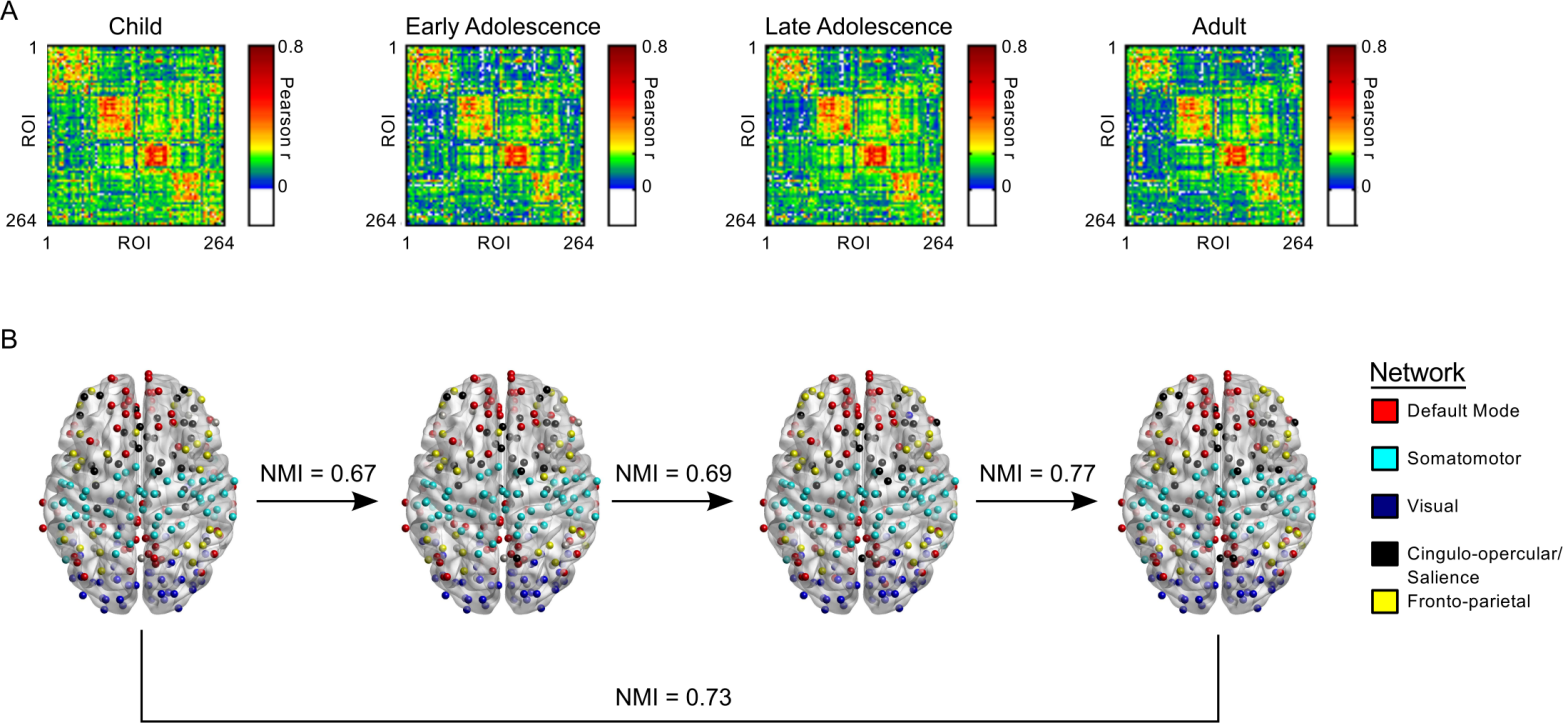
Network organization is stable prior to the onset of adolescence. (A) Group-averaged correlation matrices organized according to network affiliation. ROI order is consistent across all four groups. (B) Regions of interest imposed on a semitransparent brain. Normalized mutual information (NMI) is a measure of similarity between two sets of data. Here, NMI refers to the comparison between two sets of network affiliation vectors between each consecutive age group and between children and adults. (Data available at http://devrsfmri_2015.projects.nitrc.org/devrsfmri_2015.tar.bz2.)

We used a previously defined functional connectome parcellation of 264 functional regions of interest (ROIs) across cortical, subcortical, and cerebellar structures [14] in a sample of 192 individuals, aged 10–26 y old (Table 1). For each subject, we correlated the time series of each ROI with that of every other ROI. We then formed group matrices by averaging each subject’s connectivity matrix within categorical age groups (10–12-, 13–15-, 16–19-, and 20–26-y-olds) (See Materials and Methods) (Fig 1A). For each group, we partitioned the full functional connectome into modules using Newman’s Q-metric coupled with an efficient optimization approach [15,34,35] across network densities ranging from the top 1% to 25% of pair-wise correlations in terms of correlation strength. Notably, Newman’s Q-algorithm returns modules of densely interconnected nodes. We interpret these modules as being functionally connected collections of brain regions sub-serving common functions and therefore refer to them as functional brain networks. The representative network partition of the full connectome was given a threshold of a density of 10% (Fig 1B) to partition the network into a meaningful structure while maintaining high connectedness, which would be limited with lower thresholds. This approach identified more comprehensive networks compared with those incorporating lower thresholds [14], such that a single network encompassed the cingulo-opercular, subcortical, and salience networks. We refer to this network, which includes regions critical to cognitive control, as the CO/Salience network.

**Table 1:** Subject demographics.

Group	n	Age Mean (SD)	IQ Mean (SD)	Race	Mean FD	Mean DVARS^a^	Mean DVARS^b^
**Child**	41(20F)	11.55 (0.82)	112.10 (13.17)	28(68%) white	0.64*	26.72	2.59
**Early Adolescence**	41(18F)	14.54 (0.91)	110.17 (10.94)	30(73%) white	0.20	21.97	2.17
**Late Adolescence**	53(28F)	17.89 (0.92)	112.51 (12.01)	44(83%) white	0.22	24.84	1.60
**Adult**	57(30F)	22.38 (1.83)	116.84 (13.18)	40(70%) white	0.18	22.97	2.43

^a^ DVARS calculated prior to wavelet despiking.

^**b**^ DVARS calculated on motion time series after wavelet despiking. Large decreases indicate wavelet despiking was effective in mitigating head motion confounds.

* Mean FD was significantly greater in the child group compared to each other age group (*p* < 0.05, Tukey’s honest significant difference corrected for multiple comparisons). A one-way analysis of variance (ANOVA) was conducted between groups for mean DVARS *before* wavelet despiking (Mean DVARS ^a^) and again between groups *after* wavelet despiking (Mean DVARS ^b^), with no significant differences observed in either test (*p* > 0.05). Note FD is calculated prior to our motion correction procedure while the final DVARS values (Mean DVARs ^**b**^) are calculated after our motion correction procedure.

DVARS, root mean square derivative of fMRI timeseries; F, Female; FD, framewise displacement; SD, standard deviation.

**Fig 2:**
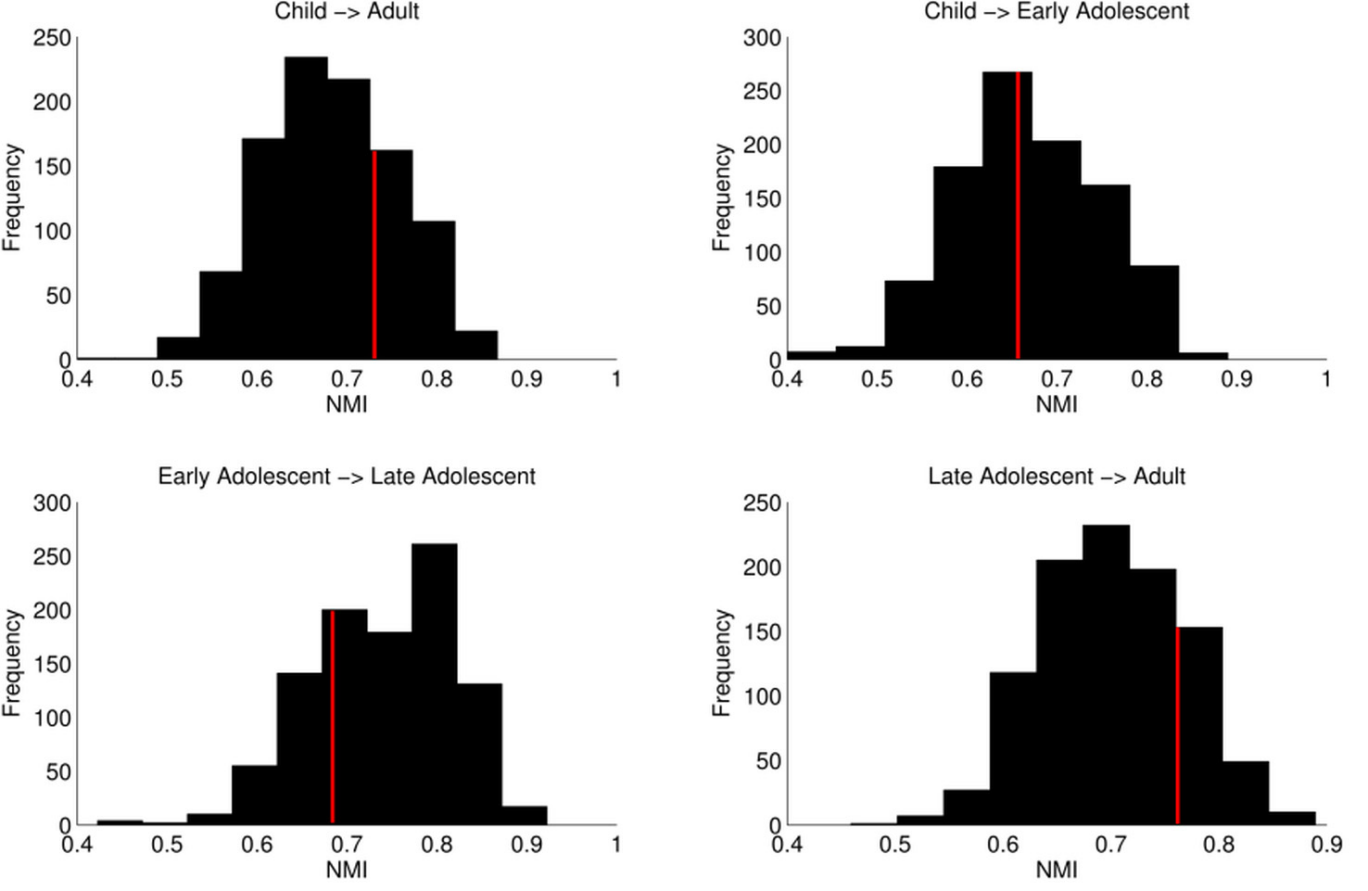
Comparison of observed NMI to a null distribution. Red lines denote the observed value for NMI. This value was plotted against a null distribution for each subsequent age group comparison and between children and adults. For each comparison, observed values fell maximally just over one standard deviation from the mean of the null distribution. Importantly, this effect was not restricted to the network density represented here (see S1 Table). (Data available at http://devrsfmri_2015.projects.nitrc.org/devrsfmri_2015.tar.bz2.)

We tested changes in network organization using normalized mutual information (NMI), which measures the mutual dependence of two variables (i.e., how much information in variable one is also contained in variable two). NMI values range from 0 to 1. A value of 0 indicates no mutual dependence (no shared information), while a value of 1 indicates complete dependency (completely shared information). We calculated NMI for networks between consecutive age groups and between children and adults (Fig 1B). We used a random permutation test to compare observed NMI values to a null distribution of 1,000 NMI values. For the adult versus child contrast, observed NMI = 0.73 (null mean [*M*] = 0.68, null standard deviation [*SD*] = 0.07); between children and early adolescents, NMI = 0.67 (null *M* = 0.73, null *SD* = 0.08); between early adolescents and late adolescents, NMI = 0.69 (null *M* = 0.76, null *SD* = 0.06); and between late adolescents and adults, NMI = 0.77 (*M* = 0.70, *SD* = 0.06) (Fig 2). Importantly, all observed NMI values fell maximally just over one standard deviation of the null mean, indicating no significant differences in network organization from late childhood into adulthood. To provide statistical evidence for findings reflecting stable network organization, we took a Bayesian approach, weighting evidence in favor of the null hypothesis (stable network organization) versus the evidence for the alternative hypothesis (dynamic network organization) [36]. First, we generated a distribution of observed NMI values by performing a leave-one-out cross validation. We removed one subject from each group for any given contrast and calculated NMI on the remaining group-averaged matrices. Then, we compared the resulting distribution to the previously generated null distribution for each contrast by calculating the Jeffreys-Zellner-Siow (JZS) Bayes factor [36]. Values greater than 1 provide evidence supporting the null hypothesis, while values between 0 and 1 provide support for the alternative hypothesis. With respect to the null hypothesis of stable developmental network organization, values ranging from 1 to 2 indicate anecdotal evidence and from 3 to 10, substantial evidence. For children versus early adolescents, JZS Bayes factor = 3.82; for early adolescents versus late adolescents, JZS Bayes factor = 2.49; for late adolescents versus adults, JZS Bayes factor = 5.34; and for children versus adults, JZS Bayes factor = 8.01. These results indicate substantial evidence in favor of stable network organization throughout late childhood, adolescence, and adulthood. Importantly, these results were robust across network densities; thus, our results were not due to our choice of representational network density (S1 Table).

In addition to group-averaged matrices, we also calculated NMI between modules defined on the basis of individual subject data and the group-averaged adult module assignments to provide an analysis of subject variability. No significant differences were observed between groups, as any potential between-group variability was found to be smaller than that of within-group variability (S1 Fig).

### Within- and Between-Network Changes in Connectivity Strength

Given network *organization* is on line by childhood and remains stable throughout this developmental period, it cannot account for cognitive changes during adolescence. Hence, we investigated developmental changes in network connectivity strength within networks (reflecting the integrity of specialized networks) and between networks (reflecting the integration of information processing across functional domains). First, we partitioned each group-averaged matrix into networks according to the adult network assignment. Consecutive age group comparisons of within- and between-network connectivity were conducted using a two-tailed *t* test that was Bonferroni corrected for multiple comparisons (*p* < 0.01).

**Fig 3:**
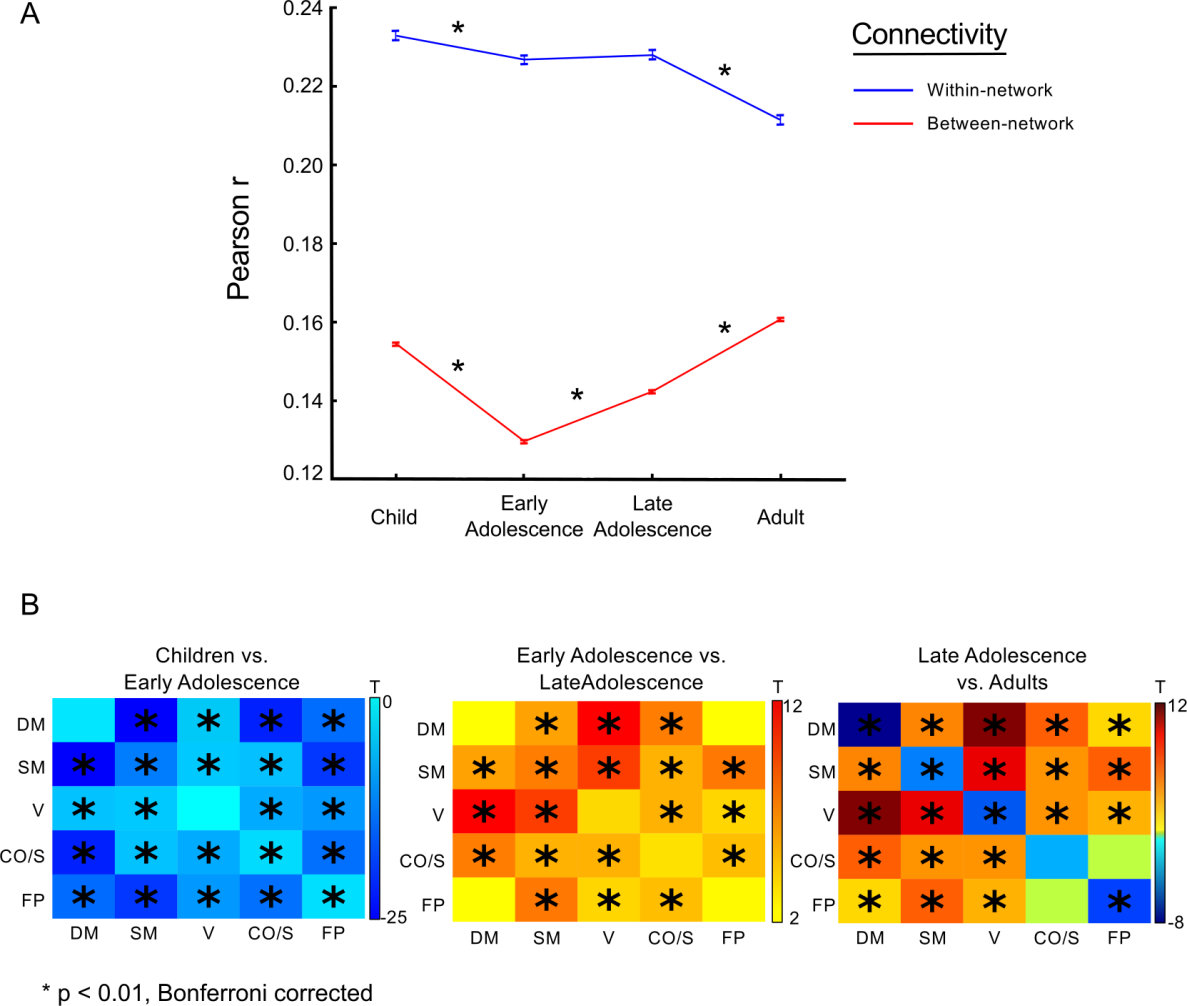
Connectivity strength changes through development as a function of network organization. (A) Connectivity strength changes as a function of within- and between-network connectivity. Asterisks denote significant differences between groups (*p* < 0.05, corrected) (B) Each cell represents the t-statistic resulting from a *t* test of connectivity strength between each network contrast. The diagonal represents within-network comparisons (e.g., DM-DM network connectivity strength differences between groups), while off-diagonal elements are between-network comparisons (e.g., DM network and CO/Salience network). Therefore, matrices are symmetric. Asterisks denote significant differences between groups (*p* < 0.01, corrected). (Data available at http://devrsfmri_2015.projects.nitrc.org/devrsfmri_2015.tar.bz2.)

Age-related changes in connectivity strength were unique to developmental stages. From childhood (10–12 y) to early adolescence (13–15 y), there was a global decrease in connectivity strength for both within-network and between-network connectivity (Fig 3A and 3B) (*p* < 0.05, corrected). From early adolescence (13–15 y) to late adolescence (16–20 y) within-network connectivity remained stable, while between-network connectivity increased across networks, with the exception of DM/FP network connectivity, which remained stable (Fig 3A and 3B). Lastly, from late adolescence (16–19 y) to adulthood (20–26 y), within-network connectivity strength again decreased, while between-network connectivity continued to increase (Fig 3A and 3B). These results indicate that the transition to adult-level network connectivity is characterized by a shift from predominance of within-network connectivity to reliance on between-network connectivity. Together, these results suggest that increased collaborative brain function may underlie improvements in cognitive control.

### No Changes in Distance-Dependent Connectivity through Adolescence

**Fig 4:**
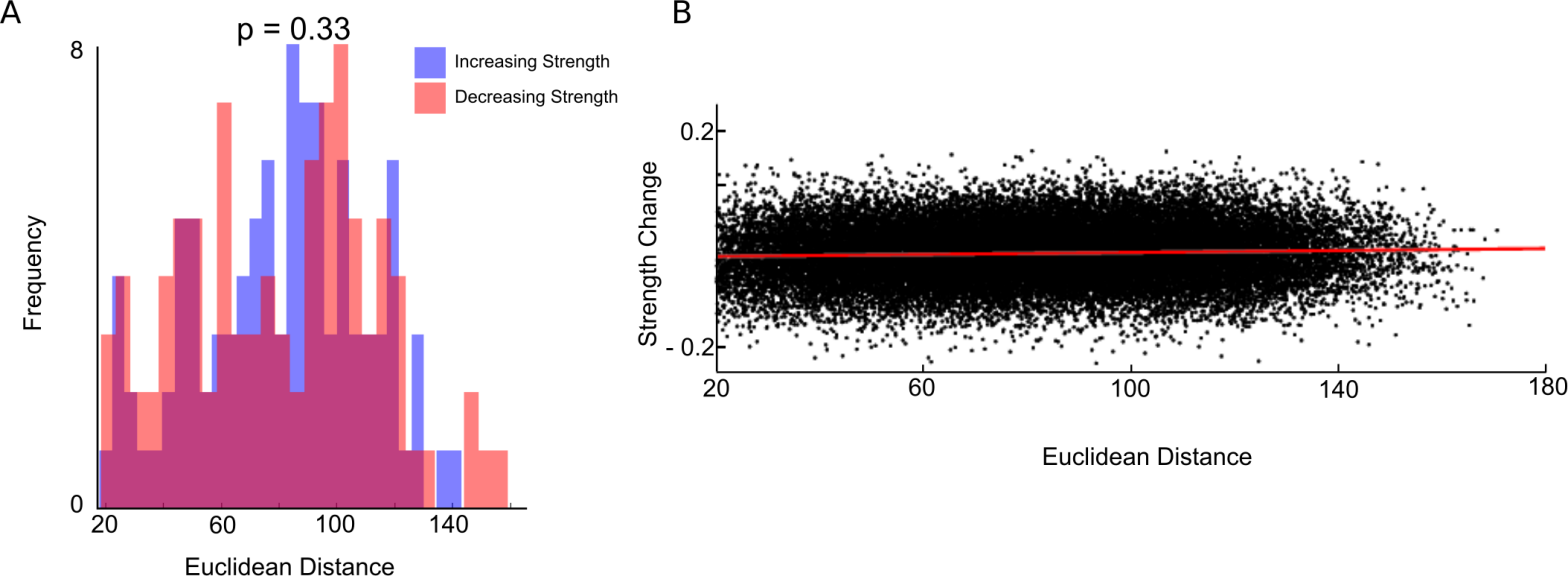
Developmental changes in connectivity strength are not a function of distance. (A) Distance distributions of significantly increasing connections (blue) and significantly decreasing connections (red) between the child and adult group. No significant difference was found between the two distributions, indicating a lack of evidence for distance-dependent effects on change in connectivity strength (*p* = 0.33). (B) Each point represents a pairwise relationship between two regions of interest. Data values represent the difference found by subtracting the averaged child matrix from the averaged adult matrix, plotted as a function of the Euclidean distance between regions of interest. No significant relationship was found between changes in correlation strength and distance (*p* > 0.05). (Data available at http://devrsfmri_2015.projects.nitrc.org/devrsfmri_2015.tar.bz2.)

Next, we examined the presence of distance-related changes with development [17,19,20]. In the present study, age-related changes in connectivity strength between ROI pairs were assessed by subtracting each pairwise relation of the averaged child connectivity matrix from the averaged adult connectivity matrix. We also calculated Euclidean distance for each pairwise relation and regressed the change in connectivity strength against Euclidean distance (Fig 4). Results showed that Euclidean distance accounted for a non-significant amount of the variance in change in connectivity with age (R^2^ = 0.002, *p* > 0.05), indicating distance alone does not play a significant role in connectivity *strength* changes from childhood to adulthood [17,19,20]. We also contrasted the distributions of the top 100 increasing and decreasing connections in terms of connectivity strength between children and adults and found no significant differences (*p* = 0.33).

### Developmental Trajectories of Network-Level Integration

**Fig 5:**
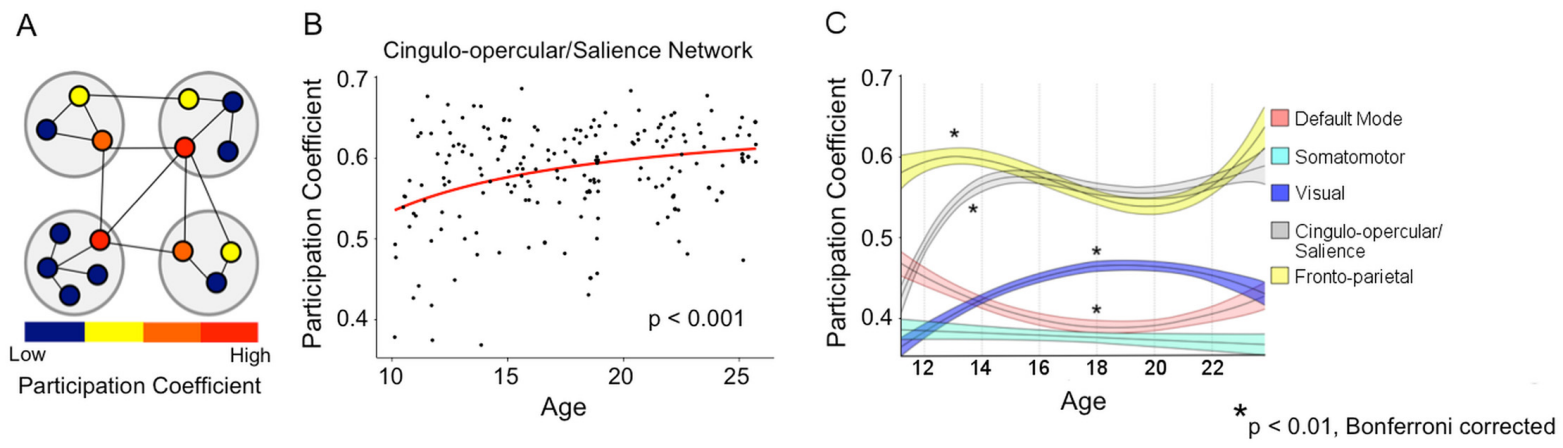
Development of network integration. (A) Model network with four communities (larger gray circles) to illustrate PC. Nodes (smaller colored circles) that are warmer colors have a larger PC due to the existence of distributed links to other networks, representing network *integration*. (B) The CO/Salience network significantly increased in PC, and thus integration, through adolescence (*p* < 0.001). No other network demonstrated any significant relationship with age in individual subjects (*p* > 0.05). (C) Development of long-term fluctuations in participation coefficient by network after smoothing data. The centerline of each curve represents the mean. Upper and lower bounds represent the 95% confidence interval. Asterisks denote statistically significant results from the regression analysis. (Data available from sheet “Fig5Fig6” in S1 Data.)

In addition to characterizing age-related changes in the strength of connectivity both as a function of network organization and as a function of distance, we also aimed to quantitatively characterize the *distribution* of these between-network interactions using graph theory. Brain regions (nodes) within networks may either contain connections (links) solely to nodes within the same network or may also contain between-network links. A node that has distributed links across multiple networks can be regarded as a highly integrated region (Fig 5A). Here, we operationally define *integration* as the level to which a region contains distributed links from its “home” network to a foreign network. Participation coefficient (PC) is a graph theoretical construct that is used to calculate integration between brain networks [25]. PC refers to the level to which a node establishes links to foreign networks, with values ranging from 0 to 1. Nodes that link solely to other nodes within their “home” network would have a PC of 0, while nodes with many distributed between-network links would have a PC closer to 1. Delineating the level of integration using a node’s PC extends beyond defining the degree (i.e., number of links) of a node, to defining the relative importance of those links with other networks [16].

To analyze developmental trajectories of integration at the network level, we calculated PC for every node within individual subject matrices at each network density. As an important aside, to remove the arbitrary bias in thresholding, all subsequent calculations involving PC are represented as the mean value across the range of network densities. Though we chose this method, PC across all nodes is significantly positively correlated with the PC of all nodes at each network density (S2 Fig). If our results were only driven by a specific threshold (e.g., 5%), but not others (e.g., 20%), a significant relationship between mean PC and the specific threshold driving the effect (5% in this example) would exist, but would not exist in others (20% in this example). This provides evidence that PC is robust to any biases that could be introduced by thresholding.

**Fig 6:**
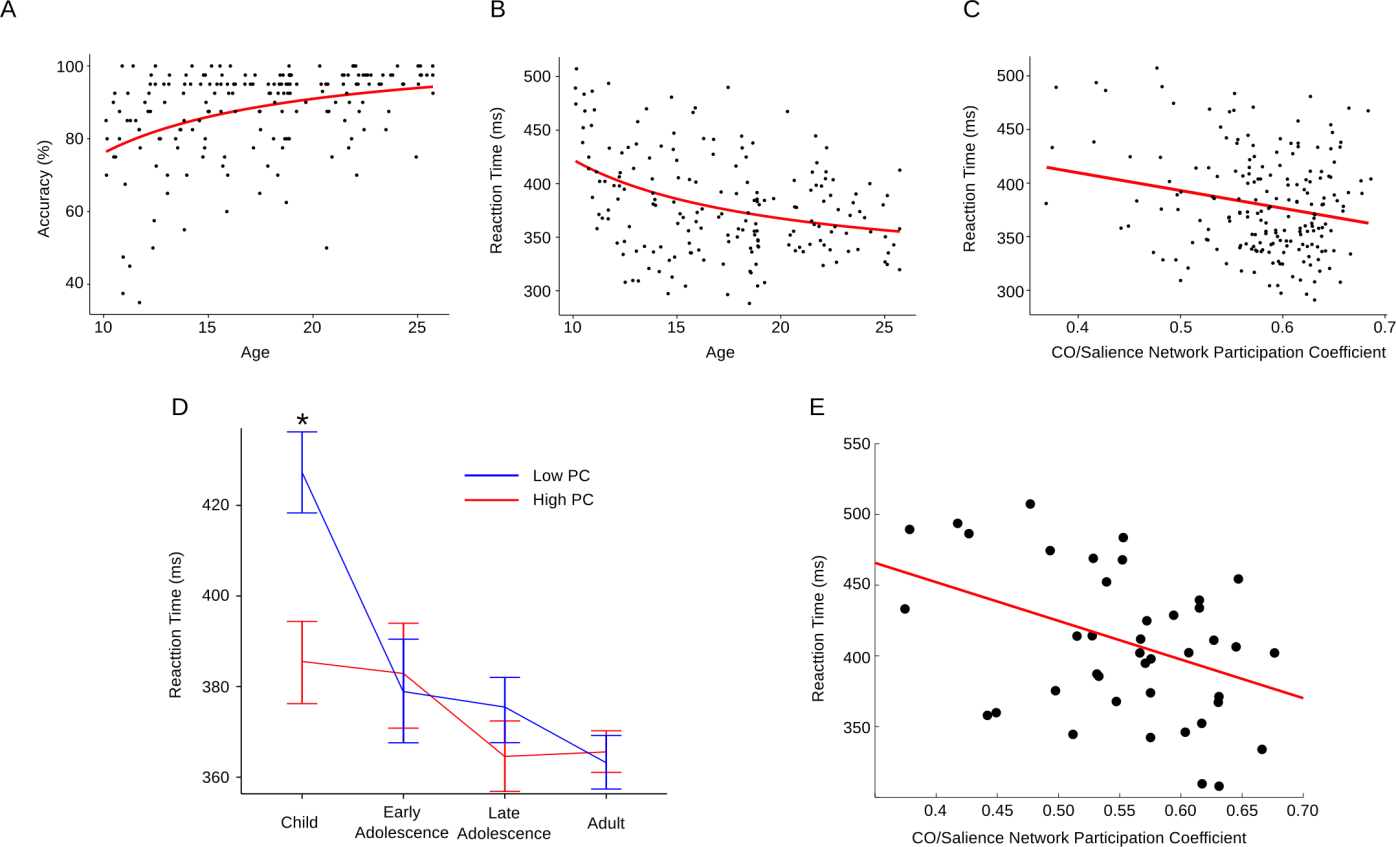
Relationship between increased cingulo-opercular/salience network integration and cognitive control. Performance on the antisaccade task improves throughout adolescence, evidenced by increased accuracy (A) and decreased reaction time (B). As integration of the CO/Salience network increases, reaction time significantly decreases (C). (D) Results from the moderation analysis. CO/Salience integration significantly moderated the effect between age and antisaccade reaction time, such that less CO/Salience integration was predictive of longer reaction times, while higher CO/Salience integration led to significantly faster reaction times (*p* < 0.001). Note that this effect only occurred during late childhood, indicating that earlier maturation of the CO/Salience network is critical for achieving adult-like behavior earlier in development. (E) Reaction time as a function of CO/Salience network integration in the child group. (Data available from sheet “Fig5Fig6” in S1 Data.)

For each subject, nodes were grouped according to the network to which they were assigned in the adult group. Then, we calculated the mean PC value for each network and tested each network for significant age-related effects on individual subjects, fitting both linear and inverse regression models, which are known to best fit this period of development [37]. The choice of superior model fit was made quantitatively, using Akaike information criterion (AIC). The PC of the CO/Salience network significantly increased over the age range studied (R^2^ = 0.09, t = 3.74, *p* < 0.001) (Fig 5B), optimally fit with an inverse model. No other network displayed age-related changes in PC for either linear or inverse models (*p* > 0.05) (Sheet “Fig5Fig6” in S1 Data). One purported role of the CO/Salience network is the maintenance of cognitive control. Thus, increased integration of the CO/Salience network with other brain networks may underlie improvements in cognitive control performance during adolescence. We tested this hypothesis by investigating associations between network integration and behavioral performance in the antisaccade task.

To identify any long-term fluctuations in PC that may not be captured at the individual subject level, we sorted individual subject matrices by age and then calculated average subject correlation matrices using a moving average approach (see Materials and Methods). After calculating PC for each region within each moving average group, we computed the mean PC within each network. We then fit linear, inverse, quadratic, and cubic regression models to the data, with the best fit model defined as the one with the lowest AIC (Fig 5C). The best fit model for the CO/Salience network was an inverse fit (R^2^ = 0.59, *p* < 0.05), showing an increase in PC from late childhood through approximately 14 y of age, followed by relative stability (Fig 5C, black curve). The quadratic model best fit age-related changes in the DM network (R^2^ = 0.28, *p* < 0.05), which decreased in PC throughout adolescence, but increased slightly into early adulthood (Fig 5C, red curve). A quadratic model best fit the visual network (R^2^ = 0.51, *p* < 0.05), with peak levels of integration occurring late in adolescence (Fig 5C, blue curve). A cubic model best fit the FP network (R^2^ = 0.29, *p* < 0.05), where PC increased from late childhood through approximately 14 y of age before declining from approximately 14 to 20 y, and then increasing again throughout early adulthood (Fig 5C, yellow curve). Lastly, the SM network remained relatively stable throughout development (R^2^ = 0.01, *p* > 0.05) (Fig 5C, cyan curve). The fact that no other network demonstrated significant age-related effects in the individual subjects analysis compared to the moving average approach suggests the lack of differences is likely due to a high amount of individual subject variability.

### Cingulo-Opercular/Salience Network Integration Moderates the Relationship between Age and Antisaccade Latency

The antisaccade task is a particularly robust test of inhibitory control that reliably shows sensitivity to cognitive development through adolescence as accuracy and reaction times (RTs) during successful response inhibition improves through adolescence [38–40]. First, we tested the effect of age on accuracy and RT separately, with age modeled as both a linear and an inverse function. As is typical for the adolescent age range [37], all regression models involving age were best fit by an inverse model, as determined by lower AIC, compared to linear models. Similar to previous studies [38–44], we found developmental increases in the accuracy of correct inhibitory response (R^2^ = 0.14, t = 5.77, *p* < 0.0000001) and decreases in RT through the adolescent period (R^2^ = 0.13, t = -5.51, *p* < 0.00001) (Fig 6A and 6B) (Sheet “Fig5Fig6” in S1 Data).

Next, we tested the association between PC of the CO/Salience network (i.e., CO/Salience network integration) and antisaccade accuracy and RT, controlling for age. Results showed no association between CO/Salience network PC and accuracy (*p* = 0.34). However, as CO/Salience network PC increased, RT to correct inhibitory responses decreased (t = -2.09, *p* = 0.03) (Fig 6C), suggesting that greater CO/Salience network integration supports timely successful inhibitory control. Notably, no other network displayed a significant relationship between PC and accuracy or RT (all *p* > 0.05).

Given the relationship between age and both antisaccade performance and CO/Salience network PC, we assessed whether CO/Salience network PC moderates the relationship between antisaccade performance and age. To test this, we ran two moderation analyses, one including CO/Salience network PC as a moderator of age and antisaccade accuracy and a second including CO/Salience network PC as a moderator of age and antisaccade RT. In each model, both regressors were centered prior to model fitting. CO/Salience network PC did not significantly moderate the relationship between age and accuracy (*p* > 0.05). However, CO/Salience network PC did moderate the relationship between age and correct antisaccade RT (R^2^ = 0.16, t = -3.28, *p* < 0.001). To identify when in development this interaction was most prominent, we investigated effects on RT within age groups by performing a median split of CO/Salience network PC (Fig 6D). We observed a significant difference in individual subjects within the child group (10–12 y) between RTs of subjects with high versus low CO/Salience network PC. Lower CO/Salience network PC resulted in slower RTs, while higher CO/Salience network PC resulted in faster RTs (t = 2.84, *p* = 0.02, Bonferroni corrected). When we extracted the data for each subject, the results showed that as PC increased, antisaccade RT decreased (R^2^ = 0.18, t = -2.99, *p* = 0.005) (Fig 6E).

### Developmental Patterns of Regional Integration

**Fig 7:**
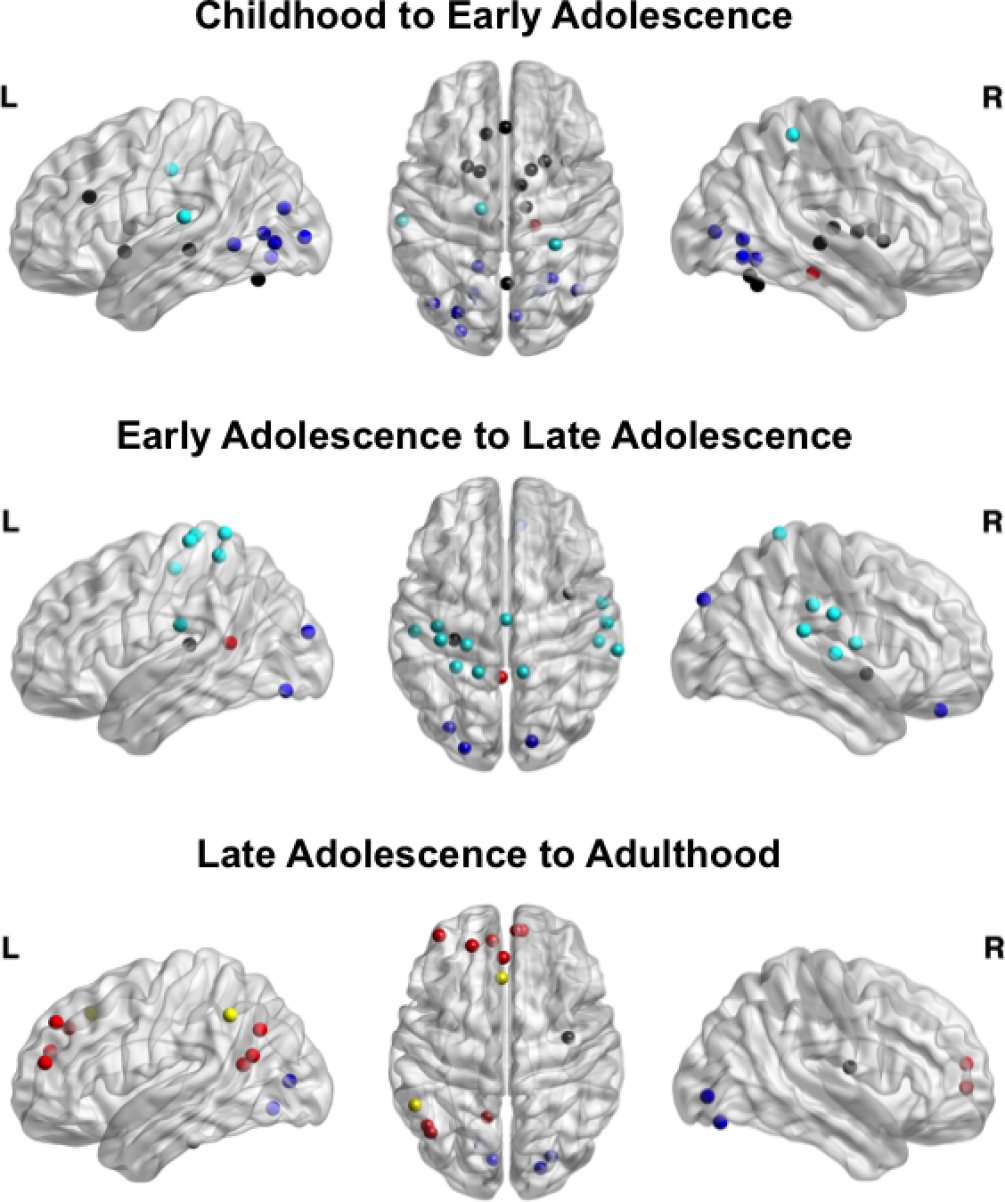
Regional increases in participation coefficient. Node color represents network affiliation as defined in Fig 1. In the transition from childhood to adolescence, most regional increases were localized to the CO/Salience network, corroborating network-level findings. During adolescence, regional increases were mostly within the SM network, while regions within the DM network and FP network increased in integration from late adolescence into early adulthood. (Data available at http://devrsfmri_2015.projects.nitrc.org/devrsfmri_2015.tar.bz2.)

In order to identify the contribution of regions of interest (ROIs) to age-related differences in network integration, which is overlooked when averaging at the network level, we tested each ROI in the network for significant increases in PC across age groups. Specifically, we permuted the connectome 1,000 times between consecutive age groups to generate null distributions for each brain region. Here, we report significant regional increases in PC in a stage-like manner throughout development (Fig 7).

#### Childhood to early adolescence

From childhood to early adolescence, 26 ROIs demonstrated significant increases in PC (Fig 7; S2 Table). Of those, two were in the DM network, three were in the SM network, ten were in the visual network, 11 were in the CO/Salience network, and zero were in the FP network. The significant increases in PC for ROIs within the SM network were mainly driven by increased degree (i.e., number of links) to the visual, CO/Salience, and FP networks, with a concomitant decrease in degree within the SM network. Within the visual network, ROIs that significantly increased in PC also increased in degree to the DM, SM, and FP networks. ROIs within the CO/Salience network showed an increase in degree with the SM, visual, and FP networks, and a decrease in degree within the CO/Salience network. Importantly, many regions within the CO/Salience network that significantly increased in PC were anatomically located in the dorsal anterior cingulate (dACC), anterior insula (aIns), and striatum, including bilateral putamen and globus pallidus.

#### Early adolescence to late adolescence

Twenty regions significantly increased in PC from early adolescence to late adolescence (Fig 7; S2 Table). Of those, two were in the DM network, 14 were in the SM network, three were in the visual network, one was in the CO/Salience network, and zero were in the FP network. Within the DM network, the posterior cingulate cortex showed a decrease in degree with the DM and visual networks, but an increase in degree to the CO/Salience network. Within the SM network, ROIs increased in both within- and between-network degree, especially to the FP, visual, and CO/Salience networks. The only region within the CO/Salience network that significantly increased in PC was the right posterior insula. This region demonstrated increased degree within network and between all networks. Three ROIs within the visual network increased significantly in PC: the left middle occipital gyrus, right cuneus, and left fusiform gyrus. All three regions increased in degree to the DM, SM, and FP networks.

#### Late adolescence to adulthood

Seventeen ROIs significantly increased in PC from late adolescence into adulthood (Fig 7; S2 Table). Of those, nine were in the DM network, one was in the SM network, four were in the visual network, one was in the CO/Salience network, and two were in the FP network. Profiles of change in degree were variable for regions within the DM network. The left superior frontal gyrus, left temporal-parietal junction (TPJ), and left fusiform all decreased in within-network degree, while the left angular gyrus, left posterior cingulate, and right medial frontal gyrus (MFG) all increased in within-network degree. The regions that increased in within-network degree also had increases in degree with other networks. The left TPJ, left angular gyrus, and bilateral MFG increased in degree to the FP network. Interestingly, many DM network regions, including the bilateral MFG, also had increased degree to the CO/Salience network. With the exception of the right lingual gyrus, the regions within the visual network that significantly increased in PC showed decreased within-network degree and increased between-network degree to each of the four other networks. For the first time throughout development, nodes within the FP network significantly increased in PC, namely the left inferior parietal lobe (IPL) and left dorsolateral prefrontal cortex (dlPFC). Both regions decreased in within-network degree and increased in between-network degree with the DM network. Additionally, the left dlPFC also decreased in degree to the CO/Salience network.

## Discussion

We sought to characterize the development through adolescence of functional brain network organization, connectivity strength, and integration. Furthermore, we tested the relationship between network integration and developmental improvements in inhibitory control. Our results provide evidence that: (1) network organization—as measured via Bayesian inference of NMI between module assignments of age groups—is stable by late childhood; (2) connectivity strength changes with development, reflecting concurrent decreases in within-network connectivity and increases in between-network connectivity; (3) anatomical distance does not account for age-related changes in connectivity strength through adolescence; (4) increased integration of the CO/Salience network occurs throughout the adolescent period; and (5) CO/Salience network PC moderates the relationship between age and antisaccade reaction time, such that higher PC, and thus integration, of this network contributes to faster RTs on the antisaccade task. These findings suggest that foundational aspects of functional network architecture, specifically network *organization*, are established early in development, while the processes underlying network *integration* continue to mature into adolescence [24]. This process reflects the way cognitive control develops, as characterized by more adaptive and flexible interactions of earlier maturing core components.

### Developmental Stability in Functional Brain Network *Organization*

Within the human functional connectome, densely interconnected brain regions are organized into well-defined functional networks, subserving sensory, motor, and cognitive functions. Our findings indicate that this network organization is stable between 10 and 26 y of age, countering earlier findings that suggested developmental changes in network organization reflect a shift from localized to distributed organization, which may have been confounded by head motion artifact [17,21,22,45,46]. The current study applied a wide array of advanced preprocessing steps to limit head motion artifact, including wavelet despiking [47], simultaneous bandpass filtering the time series data and nuisance regressors [23], as well as scrubbing [21]. These results suggest that, after controlling for head motion, there are no changes in network organization from late childhood to adulthood.

Previous studies found that many aspects of human functional network topology remain stable throughout adolescence, including small-worldness [17,18,48], global efficiency, and hub organization [24]. Combining these findings with our results showing the stability of network organization, we see strong evidence that the large-scale organization of functional networks is present by late childhood, possibly even earlier. Despite the fact the brain undergoes continual structural maturation of both gray and white matter [8,49–51], key fundamental properties of large-scale functional circuitry, including organization, are stable throughout late childhood to adulthood. While non-significant age-related changes to network organization cannot be concluded through inferential statistics, Bayesian inference via JZS Bayes factors allowed us to test the likelihood of the null versus the alternative hypothesis [36]. Using this method, we confirmed the finding that network organization does not change significantly with age.

### Age-Related Changes in Connectivity *Strength*

Our results show age-related changes in connectivity strength. Within-network connectivity strength decreased with age, suggesting that maturity results in network refinements akin to pruning unnecessary connections, which improves signal transmission within networks. On the other hand, we found between-network connectivity strength decreased into early adolescence and subsequently increased into adulthood, ultimately enhancing the ability for different networks to collaborate. Interestingly, adolescence demarcated the period when between-network connectivity began to increase, perhaps reflecting a qualitative shift in network interactions towards collaborative network functioning. The overall trend towards increased between-network connectivity is at odds with a previous study by Stevens and colleagues, who found causal between-network coupling decreased in strength [52], reflecting greater segregation of specialized networks. However, this study used an independent components analysis approach to define functional networks, which only coarsely correspond to the canonical networks used in the current study. Furthermore, this study was conducted before advances concerning mitigation of head-motion–related artifacts.

Changes in within- and between-network connectivity strength were sensitive to network organization, not solely by the distance between regions, as initial studies had suggested [17,19,20,53]. Divergences from previous results are not surprising given our implementation of recent advances in head motion control that minimized its confounds on age differences in connection strength as a function of distance [21,22]. Distance-related changes in connectivity strength by age have been found after controlling for head motion, albeit with a weaker effect than previously reported, in a sample that included children younger than those in the current sample (8 versus 10 y of age) [53]. Decreasing short-range connectivity and increasing long-range connectivity may be specific to an earlier developmental stage, when greater changes in white matter connectivity are occurring [8]. These results suggest that the adolescent transition to maturity is a period of refinements in connectivity within stable networks and concomitant increases in connectivity across widely distributed circuitry.

### Increased Integration of the Cingulo-opercular/Salience Network

While between-network connectivity increased with age, the distribution of links (i.e., integration) among networks remained stable for most networks studied. This suggests that the framework for network integration is available by childhood, with continued increases in the strength of these established between-network links. An exception, however, was the CO/Salience network, which displayed age-related changes in integration with other networks, as assessed by PC. The CO/Salience network is involved in maintaining a task set, saliency, and configuring sensory information, cognitive state, and motor output [12,54]. The continued enhancement of CO/Salience network integration follows what is known about the development of cognitive control. Core cognitive control abilities are present early in development, but the consistent successful implementation of control continues to improve into adulthood. This developmental pattern has been found for a wide range of cognitive control tasks, such as the antisaccade, go-no-go, and stroop tasks [33,55]. Our findings of stable network organization, coupled with increased integration, are consistent with these behavioral findings, suggesting that the underlying architecture supporting mature brain functioning is present early in development, with refinements continuing into adolescence.

Age differences in integration patterns at the regional scale within the CO/Salience network corroborated the network-level findings. From childhood into early adolescence, specific regions that drove increased integration of the CO/Salience network included the right aIns, bilateral dACC, anterior and mediodorsal nuclei of the thalamus, and putamen. Both the aIns and dACC are extensively anatomically connected to many major brain networks across cortical and subcortical regions [56,57]. Together these regions drive a control network guiding mental activity and behavior through an interaction of cognitive, affective, and homeostatic functions [54,58–61]. We observed an increase in the number of links between the CO/Salience network and the SM network from every region that became more integrated within the CO/Salience network, enabling more rapid access from this control system to the motor system to guide goal-directed behavior [60]. Specifically, the right aIns has been shown to play a critical developmental role as an outflow hub in directing cognitive control processes, having greater directed causal influence on other brain regions (dACC and posterior parietal cortex (PPC)) critical for proper cognitive control execution in adults compared to children. Furthermore, these functional refinements were shown to be supported structurally via enhanced white matter fiber density with development between the right aIns and PPC [62]. Additionally, it has been shown that the right aIns increases in connectivity strength to regions within network (e.g., dACC) and between networks (e.g., DLPFC and PCC), supporting its increased role in network integration over the adolescent period [63]. Due to its roles in detecting salient stimuli and acting as a switch between large-scale networks [13,61], the aIns likely plays a particularly important role in normative development, supporting enhanced integration of multiple brain processes. In addition to the right aIns, the dACC also plays a critical role in cognitive control execution [40,64]. Using a multimodal approach, Fjell and colleagues found the surface area and white matter integrity of the dACC accounted for a significant portion of variance in performance on a flanker task [65]. In sum, much like the aIns, the dACC plays a critical role in control abilities and shows a protracted development. In support of their critical developmental role, there is evidence that abnormal engagement of the aIns and dACC may underlie neurodevelopmental disorders, such as autism [53,60,66,67].

Many of the regions within the CO/Salience network that significantly increased in integrative properties were subcortical, including the putamen and thalamus. These regions show larger changes than cortical areas with respect to fractional anisotropy in white matter, increasing 30% to 50% from childhood into early adulthood [68], and also show a protracted neurophysiological development [69]. This parallels our findings of increased integration of these subcortical structures with cortical networks. Given that adolescence is a period of enhanced sensation seeking [13,37], the steep increase in the integrated nature of these regions with other brain networks during early adolescence suggests a mechanism by which motivational systems are reconfigured with more cognitive, sensory, and affective systems [70].

### Cingulo-opercular/Salience Network Integration Moderates Age-Related Improvements in Inhibitory Control

In agreement with an extensive literature [33,40], we found age-related decreases in reaction times of correct inhibitory responses. Our network analyses indicated that increased CO/Salience network integration predicted faster RTs on the antisaccade task, underscoring the importance of the CO/Salience network integrating with other networks, subserving cognitive control. Importantly, we found that CO/Salience network integration moderated decreases in antisaccade latency as a function of age. This moderation was significant in the transition from late childhood to early adolescence, when (at both the network and the regional scale) the CO/Salience network became significantly more integrated with other functional networks. Together, these results indicate that development brings greater integration between the CO/Salience network, supporting sustained cognitive control [12], and regions that underlie action such as the SM network, resulting in the ability to generate quicker execution of correct cognitive control signals [64].

### The Role of Intrinsic Functional Couplings in Integration

Although intrinsic, spontaneous coupling between regions at frequencies <0.1 Hz has been studied for nearly 20 y, the neural substrate and the meaning of the slow frequency signal remains unclear [71,72], though functional networks observed using fMRI have also been identified using magnetoencephalography [73]. Many ROI-ROI pairs demonstrate high correlations between their time courses despite a lack of monosynaptic connections [74,75]. Though the functional purpose of spontaneous slow frequency BOLD oscillations is not known, a range of possibilities exist. Resting-state functional networks may be groups of regions that often coactivate in task-based settings, reflecting a history of coactivation [12,76,77]. This interpretation is supported by studies finding strong resting-state correlations, despite the lack of a direct anatomical connection. However, the existence of strong functional connectivity in the absence of direct anatomical connections allows for other alternatives, including the notion that resting-state networks are constantly sampling a possibility of configurations, constrained by anatomy, to make predictions about optimal network configurations for a given input [72]. Furthermore, over long timescales, such as in this study, resting-state functional brain networks are dependent on anatomical connectivity; however, at shorter timescales, numerous configurations are possible [78]. That said, changes in the framework of integration within the functional connectome during adolescence may reflect differences in the pattern in which information is shared across distributed neural networks. Specifically, from a graph theoretic view, the regions that significantly increased in participation coefficient are areas that integrate across multiple functional networks to a greater extent. Importantly, the role these brain regions play in integrating information may reflect a particular vulnerability for the emergence of psychopathology, which emerges during adolescence—a time when the brain is reorganizing the way it shares and processes information across these networks.

### Limitations

This study was not without limitations. The sample was cross-sectional, undermining our ability to analyze subject-specific growth trajectories. We are also limited by some inherent drawbacks of fMRI, including residual head motion, though we took multiple processing steps towards mitigating these effects, including wavelet despiking, simultaneous bandpass filtering of the time series and nuisance regressors, and scrubbing. Additionally, 5 min of resting-state data is considered a minimum requirement for analyses of resting-state fMRI data, with recent pushes for longer acquisitions [75,79]. However, longer acquisitions may lead to even greater differences between age groups in head motion. Lastly, because PC was averaged over all nodes within a network, it is possible that some single brain regions could be driving this effect more than others. That said, we still found CO/Salience network increases in integration with age that moderated the relationship between cognitive control performance and age. This finding stresses the importance of network integration for adult-like cognitive control performance, rather than the maturation of any singular brain region. Future studies could aim to elucidate specific brain regions driving cognitive control maturation via integration.

## Materials and Methods

### Participants

One hundred and ninety-five subjects aged 10–26 y participated in this study (Table 1). Written informed consent was obtained from every subject and minors did sign assents. This research was approved by the University of Pittsburgh Institutional Review Board. A phone screen questionnaire was used to assess medical history and history of psychiatric disorders at the time of recruitment. Subjects were excluded at the time of recruitment if the subject or a first-degree relative currently or previously had a psychiatric disorder. Subjects also completed a battery of self-report measures of psychopathology. As determined through the interview process, neither subjects included in this study nor their first-degree relatives currently or previously had any neurological disease, brain injury, or diagnosed psychiatric illness. Substance use was assessed using the drug use and history questionnaire. Subjects included in this study were free from substance use or abuse. A post-scan questionnaire was used to inquire if subjects had fallen asleep. Sixteen subjects reported periods when they may have briefly drifted into sleep but none reported sleeping throughout the entire resting state scan. Data from three subjects were discarded due to excessive head motion. Therefore, we report data from 192 subjects. While age was considered as a continuous variable, some analyses considered developmental stages by binning ages after first sorting individual subjects by age, similar to methods used in the past to characterize changes in childhood (*n* = 41 10–12 y olds), early (*n* = 41 13–15 y olds) and late adolescence (*n* = 53 16–19 y olds), and adulthood (*n* = 57 20–26 y olds).

### Antisaccade Task

The antisaccade task was performed by subjects outside of the MR scanner on a separate day from the MR visit. For a full description of the antisaccade task used, see [80]. Briefly, neutral trials were extracted from an incentivized antisaccade task, consisting of reward, loss, and neutral trials. There were a total of 40 of each trial type. Each neutral trial began with a white central fixation, which then turned red for 1.5 s, prompting subjects to prepare a response. Next, a peripheral stimulus (yellow dot at approximately 0.5 degree/visual angle) appeared at an unpredictable location on the horizontal meridian (±4 and 8 degrees/visual angle) for 1.5 s. Subjects were instructed to inhibit making a saccade towards the stimulus, and instead to saccade to the mirror location of the stimulus. Eye movement data were scored online using interfaced E-Prime (Psychology Software Tools, Inc., Pittsburgh, PA) and ASL (Applied Science Laboratories, Bedford, MA) eye tracking software. A script detected if at any time during the first 1,000 ms a subject made a saccade to the stimulus or if no eye movement was generated. An auditory tone (1,163 Hz) was played for 400 ms if the subject made a saccade to the stimulus. If the subject made a correct saccade a “cha-ching” sound (1,516 Hz) was presented for 400 ms. Correct responses were defined as those in which the first eye movement in the saccade was directed toward the mirror location at a velocity greater than or equal to 30°/s [81] and extended beyond a 2.5°/visual angle from the central fixation. A response was considered incorrect when the first saccade was directed towards the target beyond a 2.5°/visual angle from central fixation, but were subsequently directed to the hemifield opposite the target, similar to previously published work [80].

### Eye Tracking

In addition to the online scoring, eye data were scored offline by a technician for various saccade metrics, including correct trials and errors, as well as saccade latency, using ILAB software [81] and an in-house scoring suite written in MATLAB (Math Works, Inc., Natic, MA). A correct antisaccade response was one in which the first saccade following stimulus onset was towards the mirror location of the stimulus and extended beyond a 2.5 degrees/visual angle central fixation zone. Errors were defined as occurring when the first saccade following stimulus onset was directed towards the stimulus and extended beyond central fixation.

### MR Data Acquisition

Data were acquired using a 12-channel Siemens 3T Tim Trio at the University of Pittsburgh Medical Center Magnetic Resonance Research Center. The resting-state scan was acquired at the end of the scanning session and was always at the same time of acquisition for all subjects. For each subject, we collected 300 s (200 TRs) of resting-state data. Structural images were acquired using a sagittal magnetization-prepared rapid gradient-echo sequence (repetition time [TR] = 1,570 ms, echo time [TE] = 3.04 ms, flip angle = 8°, inversion time [TI] = 800 ms, voxel size = 0.78125 × 0.78125 × 1 mm). Functional images were acquired using an echo-planar sequence sensitive to BOLD contrast (T_2_*; TR = 1.5 s, TE = 29 ms, flip angle = 70°, voxel size = 3.125 × 3.125 mm in-plane resolution, 29 contiguous 4-mm axial slices). During the resting-state scan, subjects were asked to close their eyes and relax, but not fall asleep.

### RS-fMRI Preprocessing

Functional images were preprocessed using AFNI [82] and Freesurfer [83]. Standard preprocessing steps were completed, including (1) normalization based on global mode, (2) wavelet despiking [47], (3) simultaneous multiple regression of nuisance variables from BOLD data and bandpass filtering [23] at 0.009 Hz < *f* > 0.08, and (4) spatial smoothing using a 6 mm full-width at half-maximum Gaussian blur. Freesurfer was used to segment gray matter, white matter, and ventricular voxels. Nuisance regressors included ventricular signal averaged from ventricular regions of interest (ROIs), six head realignment parameters obtained by rigid body head motion correction, and the derivatives of these signals and parameters. In addition to wavelet despiking, we removed any remaining high motion volumes via a scrubbing procedure [21,22]. For the original 195 subjects, we calculated two quality control measures with respect to head motion, volume-to-volume framewise displacement (FD) and the root mean square derivative of fMRI timeseries (DVARS). We censored and removed volumes in individual subjects that had an FD > 0.5 mm and DVARS > 5, as well as the frame preceding the motion artifact and the two subsequent frames. FD is calculated on the original motion time series (i.e., before motion correction with wavelet despiking). On the other hand, DVARS is calculated after motion correction with wavelet despiking. Large DVARS values after wavelet despiking would indicate motion/artifact-related noise in the global signal (i.e., brain-wide change from one volume to the next) still remained after despiking, which we did not observe (Table 1: note DVARS after wavelet despiking is considerably lower in all four groups than DVARS calculated prior to wavelet despiking). Because we collected 300 s of data, subjects were dropped entirely if >20% of their volumes were removed, leaving the minimum amount of rest data for any subject 240 s. This procedure resulted in the removal of three subjects from further analyses. Of the remaining 192 subjects, only four did not contain a full 300 s of data.

### Functional Network Parcellation

For each subject, nodes (*n* = 264) were defined from the functional parcellation defined by Power and colleagues [14]. Coordinates were derived through fc-Mapping [84,85] and a meta-analytic procedure [14], covering major brain systems involved in both tasks and rest. All ROIs were modeled as 10 mm diameter spheres around a center coordinate. For each subject, the timeseries of voxels within each ROI were averaged and then correlated to produce a 264 × 264 correlation matrix. Any comparisons made between correlations were transformed to z values using Fisher z(r) transformation, and then reconverted to Pearson r values for reporting and visualization.

### Individual and Group Correlation Matrices

Network-level age-related changes were assessed using individual correlation matrices. For all other RS-fMRI analyses, age was treated as a categorical variable to assess stage-like developmental changes in graph metrics and changes in the distribution of connections between children (aged 10–12), early adolescents (aged 13–15), old adolescents (aged 16–19), and adults (aged 20–26). Notably, no standard for binning age groups over adolescence currently exists, though binning roughly follows Luna and colleagues [37]. Since short-distance correlations (Euclidean distance <20mm) can arise from artifacts [21], these connections were not included in tests for age-dependent significant strength changes in connectivity.

### Network Detection and Comparison

Since there is no ideal, biologically salient threshold that definitively defines functional networks, we explored a range of network densities from 1%–25% to avoid any thresholding bias. Results involving PC at the group level reflect values that are averaged across all network densities to remove any bias of a single threshold. For a representative network assignment, we chose a network density of 10%, since this threshold results in meaningful network organization (i.e., five networks), while maintaining full connectedness. Importantly, we did not impose network assignments according to [14], since that would erode the ability to make conclusions concerning developmental changes in network organization.

To define and examine the developmental trajectory of functional network organization, we partitioned the full connectome of 264 ROIs into modules using Newman’s Q-metric coupled with an efficient optimization approach proposed by Blondel et al. [15,34,35]. This method has been verified to be one of the best-performing community detection algorithms of undirected networks [86]. We then calculated normalized mutual information (NMI) to determine the level of similarity between network assignments across age groups, with values closer to 0 indicating dissimilar network assignments and values closer to 1 indicating similar assignments. NMI is a standard measure for assessing the degree of similarity between two distributions, which has been used to compare sets of network assignments in resting-state fMRI data [16,21]. NMI measures information shared between two probability distribution functions, specifically measuring how much knowing one distribution leads to certainty of the other. Furthermore, NMI will detect any type of relationship between two distributions, making it more robust than a simple correlation coefficient. In this way, we can empirically test the level of similarity of these distributions across subjects. To this end, we permuted the labels of individual matrices between contrasts 1,000 times to generate a null distribution of NMI values for each contrast. Matrices between groups were randomly shuffled and partitioned into functional networks, and NMI was calculated. Upon the finding that the observed NMI values fell around one standard deviation of the mean of the null distribution, we executed a leave one out cross validation to generate a distribution of observed NMI values for the following analysis. Because conventional significance testing does not allow stating evidence in favor of null findings, we implemented a Bayes factor alternative [36] to compare the observed NMI distribution with the null distribution. Values greater than 1 indicate the likelihood of stable functional network organization is “n” times more likely than the likelihood of developmental changes in functional network organization.

### Connectivity Strength Changes during Adolescence

A general concept in the development of functional networks is that they develop from “local to distributed” [17]. To test this hypothesis, given methodological improvements for head motion and a denser, more representative functional network [14], we contrasted connectivity values from averaged weighted matrices in children versus adults for each ROI-ROI pair. Euclidean distance was also calculated for each pairwise relation. We then performed a simple linear regression with distance as a predictor of change in connectivity strength between the children and adult matrices.

We also addressed changes in connectivity strength as a function of within- and between-network interactions. First, within each group-averaged matrix, we averaged all within-network pairwise relations and all between-network pairwise relations, separately. We then performed a two-tailed *t* test for each consecutive age contrast. We then wanted to test for significant increases or decreases in connectivity with respect to specific network interactions. To this end, within each group-averaged matrix, the average connectivity strength was calculated for each network. We then tested each combination of within-network (e.g., DM/DM network) and between-network (e.g., DM/FP networks) interactions to determine significant increases or decreases in connectivity strength between consecutive age groups. For each comparison, we ran a two-tailed *t* test to determine significance (Bonferroni corrected for multiple comparisons).

### Developmental Changes in Participation Coefficient at the Network-level

For each subject, we partitioned the full network into sub-networks imposing the module assignments from the adult group in the analysis outlined above, and subsequently calculated PC for every node within each group. PC is a graph measure quantifying the degree to which a node engages in inter-network communication [25,26]. Higher PC indicates more distributed between network connectivity, while a PC of 0 signifies a node’s links are completely within its home network (within network). Nodal PCs were then averaged within each network and were tested for significant age-related effects using linear and inverse models.

### Long-Term Fluctuations in Network-Level Participation Coefficient

To determine any long-term fluctuations in PC that may not be captured at the individual subject data, we calculated average subject correlation matrices using a moving average approach, used previously in functional brain network data [17] and commonly used in economics research. Averaged group matrices were formed using a moving average of age-ordered subjects (e.g., group1: subjects 1–30, group2: subjects 2–31, … group163: subjects 163–192), thus generating 163 groups of 30 subjects in each group. Each group matrix was then parcellated according to the adult network assignment and PC was calculated for each ROI within each group. For each group, the PC for ROIs within a network were averaged and plotted as a function of age.

### Relating Changes in Integration to the Development of Inhibitory Control

To test the hypothesis that the relationship between age and performance (accuracy and RT) on the antisaccade task is moderated by integration of the CO/Salience network with other functional networks, a hierarchical multiple regression analysis was conducted separately for accuracy and reaction time. If a significant interaction was observed, age groups were binned into the four age groups previously defined and a median split of the averaged PC within the CO/Salience network was conducted. Within each bin, we tested for significant differences in RT using a *t* test between high and low PC groups and corrected for multiple comparisons using the Bonferroni method.

### Identifying Specific Nodes Increasing in Participation Coefficient

We sought to discover brain regions that significantly increased in the ability to integrate information from widespread functional networks using graph theory. PC was calculated for each node within each categorical age group. Importantly, the degree, or number of links a node has, was not considered as a metric for integration since network measures that are degree-based have recently been called into question in Pearson correlation RS-fMRI networks [26]. PC for each node was contrasted between each set of chronological age groups (children versus early adolescents, early adolescents versus late adolescents, and late adolescents versus adults) and between adults and children by subtracting the younger group’s PCs from the older group’s PCs resulting in four total contrasts. Permutation tests were conducted on each node to test nodes for significant changes in PC. To generate a null distribution of PCs for each node, subject labels were randomized within groups 1,000 times and PC was calculated for every node in each run. Contrasts between age groups were then generated by subtracting the PCs for each node for the younger group from the older group. This process was repeated for each age contrast. A significant increase or decrease in participation coefficient for a node was Bonferroni corrected for multiple comparisons.

### Age-Related Changes in the Distribution of Regional Participation Coefficient

Within each group, and for each node that significantly increased in PC, we calculated the degree of the ROI to each network, including its “home” network, and then contrasted these values for consecutive age groups for comparison. The degree of a node is determined by the number of links a node has. This approach allowed us to contrast the distribution of links to each network between consecutive age groups (i.e., within-network versus between-network connectivity), giving us the ability to characterize the driving factor(s) behind the observed significant increases in PC.

### Computations and Visualizations

AFNI [82] and Freesurfer [83] were used to process MRI images. We used the Brain Connectivity Toolbox [28] in MATLAB (The Mathworks, Natick, MA) for network computations and statistical testing. For brain visualizations, we used the BrainNet Viewer [87].

### Conclusion

These results provide evidence that the period of childhood through adulthood is characterized by increased integration of widely distributed but stable networks. As such, a critical component underlying the adolescent transition to adult-level execution of control is the refinement and strengthening of integration between existing specialized networks. In particular, the CO/Salience network continues to increase its integration with and, thus, its influence on other networks, providing a mechanism for developmental improvements in cognitive control. These findings support a novel two-stage model of adolescent brain development in which network organization stabilizes prior to adolescence, while the integration of information across widely distributed circuitry increases in the transition from adolescence to adulthood.

## Supporting Information

S1 Data Antisaccade data provided for Fig 5 and Fig 6. (XLSX)

S1 Fig Normalized mutual information between individual subjects and adults. (TIFF)

S2 Fig Participation coefficient is robust to network density. *Y*-axis represents participation coefficient (PC) for the representative network density. *X*-axis represents mean PC across network densities.
(TIFF)

S1 Table Stable network organization is not dependent on network density. (DOCX)

S2 Table Regional increases in participation coefficient. DM = default mode network; SM = somatomotor network; Vis = visual network; CO/S = cingulo-opercular/salience network; FP = fronto-parietal network; w/in = degree change in within-network connectivity; b/w = degree change in between-network connectivity. Each cell within each of these columns represents the change in degree.
(DOCX)

## Abbreviations

AIC: Akaike information criterion
aIns: anterior insula
CO/Salience: cingulo-opercular/salience
dACC: dorsal anterior cingulate cortex
dlPFC: dorso-lateral prefrontal cortex
DM: default mode
DVARS: root mean square derivative of fMRI timeseries
FD: framewise displacement
FP: fronto-parietal
IPL: inferior parietal lobe
JZS: Jeffreys-Zellner-Siow
MFG: middle frontal gyrus
NMI: normalized mutual information
PC: participation coefficient
PPC: posterior parietal cortex
RS-fMRI: resting state functional magnetic resonance imaging
ROI: region of interest
RT: reaction time
SM: somatomotor
TPJ: temporal-parietal junction
