# Supplementary figures and images for: The Contribution of Network Organization and Integration to the Development of Cognitive Control
Source: PLoS Biol. 2015 Dec 29;13(12):e1002328. doi: 10.1371/journal.pbio.1002328 (PMC4694653; doi:10.1371/journal.pbio.1002328)

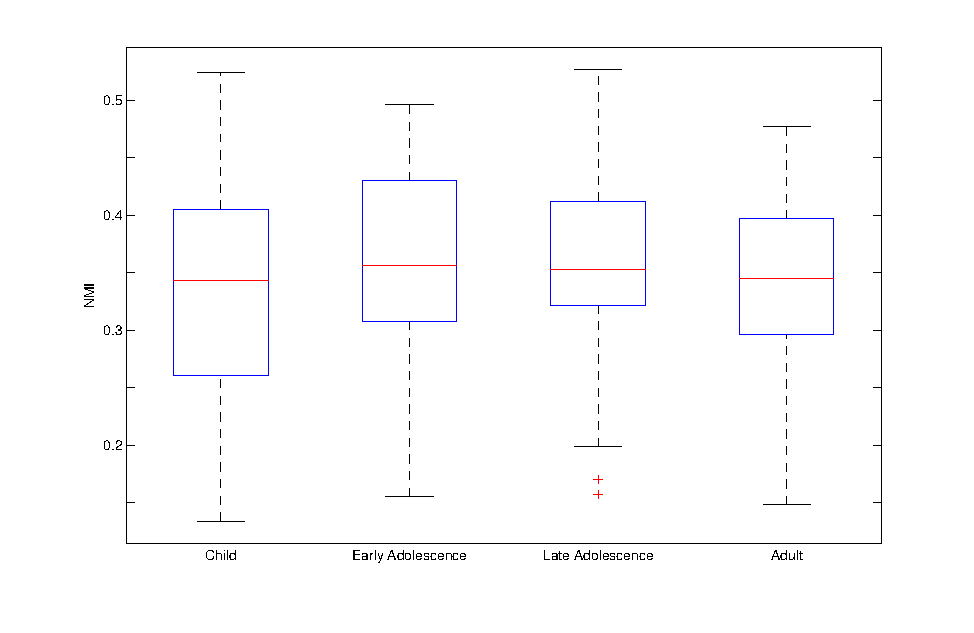

Supplement: S1 Fig — (TIFF) [file pbio.1002328.s002.tiff]

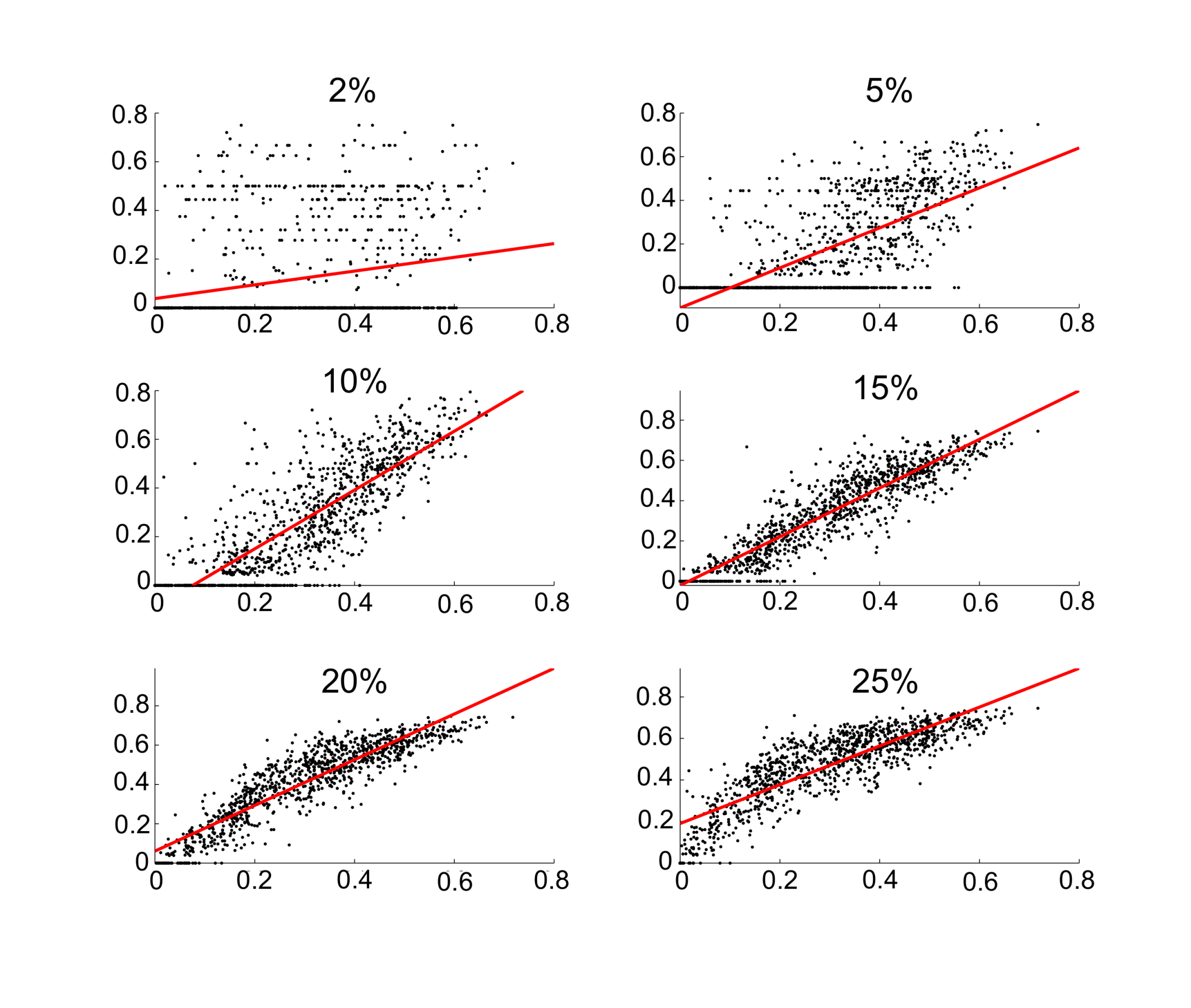

Supplement: S2 Fig — Y-axis represents participation coefficient (PC) for the representative network density. X-axis represents mean PC across network densities. (TIFF) [file pbio.1002328.s003.tiff]
