# Supplementary material for: The Contribution of Network Organization and Integration to the Development of Cognitive Control
Source: PLoS Biol. 2015 Dec 29;13(12):e1002328. doi: 10.1371/journal.pbio.1002328 (PMC4694653; doi:10.1371/journal.pbio.1002328)
Supplement: S1 Table — (DOCX) [file pbio.1002328.s004.docx]

| **Network Density (%)** | **Adult vs. Child** | | **Child vs. Early Adolescence** | | **Early Adolescence vs. Late Adolescence** | | **Late Adolescence vs. Adult** | |
| --- | --- | --- | --- | --- | --- | --- | --- | --- |
|  | Observed | Null (mean) | Observed | Null (mean) | Observed | Null (mean) | Observed | Null (mean) |
| 1 | 0.43 | 0.49 | 0.53 | 0.49 | 0.48 | 0.49 | 0.42 | 0.5 |
| 2 | 0.33 | 0.37 | 0.56 | 0.48 | 0.58 | 0.39 | 0.35 | 0.39 |
| 3 | 0.4 | 0.45 | 0.47 | 0.4 | 0.51 | 0.4 | 0.34 | 0.4 |
| 4 | 0.52 | 0.53 | 0.53 | 0.46 | 0.54 | 0.47 | 0.49 | 0.46 |
| 5 | 0.5 | 0.58 | 0.54 | 0.61 | 0.56 | 0.64 | 0.54 | 0.6 |
| 6 | 0.63 | 0.62 | 0.67 | 0.64 | 0.7 | 0.64 | 0.63 | 0.61 |
| 7 | 0.62 | 0.65 | 0.67 | 0.67 | 0.65 | 0.65 | 0.61 | 0.63 |
| 8 | 0.63 | 0.66 | 0.67 | 0.64 | 0.65 | 0.6 | 0.59 | 0.61 |
| 9 | 0.69 | 0.67 | 0.51 | 0.65 | 0.49 | 0.61 | 0.6 | 0.66 |
| 10 | 0.73 | 0.68 | 0.67 | 0.66 | 0.69 | 0.65 | 0.77 | 0.7 |
| 11 | 0.74 | 0.68 | 0.55 | 0.71 | 0.57 | 0.71 | 0.72 | 0.75 |
| 12 | 0.8 | 0.68 | 0.7 | 0.7 | 0.77 | 0.72 | 0.81 | 0.75 |
| 13 | 0.72 | 0.69 | 0.69 | 0.71 | 0.76 | 0.71 | 0.72 | 0.72 |
| 14 | 0.65 | 0.69 | 0.69 | 0.72 | 0.73 | 0.71 | 0.69 | 0.68 |
| 15 | 0.63 | 0.69 | 0.68 | 0.7 | 0.63 | 0.68 | 0.72 | 0.67 |
| 16 | 0.64 | 0.69 | 0.61 | 0.71 | 0.65 | 0.68 | 0.63 | 0.67 |
| 17 | 0.8 | 0.69 | 0.69 | 0.71 | 0.71 | 0.78 | 0.8 | 0.68 |
| 18 | 0.74 | 0.7 | 0.66 | 0.74 | 0.75 | 0.7 | 0.78 | 0.71 |
| 19 | 0.75 | 0.7 | 0.67 | 0.74 | 0.74 | 0.71 | 0.79 | 0.71 |
| 20 | 0.72 | 0.7 | 0.67 | 0.73 | 0.73 | 0.71 | 0.73 | 0.72 |
| 21 | 0.71 | 0.7 | 0.68 | 0.71 | 0.74 | 0.71 | 0.75 | 0.7 |
| 22 | 0.72 | 0.7 | 0.66 | 0.7 | 0.7 | 0.72 | 0.67 | 0.7 |
| 23 | 0.71 | 0.7 | 0.64 | 0.7 | 0.7 | 0.73 | 0.74 | 0.7 |
| 24 | 0.67 | 0.7 | 0.61 | 0.71 | 0.7 | 0.75 | 0.7 | 0.72 |
| 25 | 0.73 | 0.7 | 0.61 | 0.71 | 0.72 | 0.76 | 0.76 | 0.72 |
