# Supplementary material for: The Contribution of Network Organization and Integration to the Development of Cognitive Control
Source: PLoS Biol. 2015 Dec 29;13(12):e1002328. doi: 10.1371/journal.pbio.1002328 (PMC4694653; doi:10.1371/journal.pbio.1002328)
Supplement: S2 Table — DM = default mode network; SM = somatomotor network; Vis = visual network; CO/S = cingulo-opercular/salience network; FP = fronto-parietal network; w/in = degree change in within-network connectivity; b/w = degree change in between-network connectivity. Each cell within each of these columns represents the change in degree. (DOCX) [file pbio.1002328.s005.docx]

| **Contrast** | **ROI** | **Hemisphere** | **Network** | **Δ Degree** | **DM** | **SM** | **Vis** | **CO/S** | **FP** | **w/in** | **b/w** |
| --- | --- | --- | --- | --- | --- | --- | --- | --- | --- | --- | --- |
| **Childhood vs. Early Adolescence** | Culmen | R | DM | 2 | 0 | 0 | 2 | 0 | 0 | 0 | 2 |
|  | Precuneus | L | SM | 2 | 1 | -4 | 3 | 1 | 1 | -5 | 7 |
|  | Postcentral G. | R | SM | 1 | -1 | -5 | 4 | 1 | 2 | -5 | 6 |
|  | Insula | R | CO/S | 14 | 2 | 9 | 2 | 1 | 0 | 1 | 13 |
|  | Postcentral G. | L | SM | 17 | 3 | -1 | 11 | 3 | 2 | -1 | 18 |
|  | Middle Occipital | L | VIS | 16 | 6 | 5 | 0 | 5 | 0 | 0 | 16 |
|  | Lingual | R | VIS | 12 | 1 | 8 | 1 | 0 | 2 | 1 | 11 |
|  | Parahippocampal G. | R | VIS | 11 | 0 | 8 | 2 | 0 | 1 | 2 | 9 |
|  | Lingual | L | VIS | 4 | 2 | 2 | -1 | 0 | 1 | -1 | 5 |
|  | Cuneus | L | VIS | 23 | 3 | 8 | 6 | 3 | 3 | 6 | 17 |
|  | Lingual | L | VIS | 5 | 2 | 1 | 1 | 0 | 1 | 1 | 4 |
|  | Middle Occipital | R | VIS | 9 | 2 | 5 | 0 | 0 | 2 | 0 | 9 |
|  | Middle Occipital | L | VIS | 9 | 3 | 4 | 0 | 0 | 2 | 0 | 9 |
|  | Cuneus | R | VIS | 6 | 3 | 2 | -2 | 0 | 3 | -2 | 8 |
|  | Middle Occipital | L | VIS | 8 | 2 | 3 | 2 | 0 | 1 | 2 | 6 |
|  | Anterior Cingulate | L | CO/S | -5 | 0 | 1 | 0 | -6 | 0 | -6 | 1 |
|  | Anterior Cingulate | L | CO/S | 1 | 1 | 1 | -1 | -3 | 3 | -3 | 4 |
|  | Thalamus | R | CO/S | -6 | 1 | 1 | 1 | -8 | -1 | -8 | 2 |
|  | Thalamus | R | CO/S | 1 | -3 | 4 | 0 | -5 | -6 | -5 | 6 |
|  | Thalamus | L | CO/S | 0 | 2 | 1 | 2 | -5 | 0 | -5 | 5 |
|  | Lentiform | L | CO/S | 12 | 2 | 6 | 1 | 3 | 0 | 3 | 9 |
|  | Lentiform | L | CO/S | 21 | 6 | 7 | 0 | 3 | 5 | 3 | 18 |
|  | Lentiform | R | CO/S | 2 | 0 | 4 | 0 | -1 | -1 | -1 | 3 |
|  | Lentiform | R | CO/S | 4 | 1 | 3 | 1 | -1 | 0 | -1 | 5 |
|  | Thalamus | R | CO/S | 6 | 7 | 1 | -1 | -6 | 5 | -6 | 12 |
|  | Declive | L | DM | 12 | 0 | 6 | -1 | 7 | 0 | 0 | 12 |
| **Early Adolescence vs. Late Adolescence** | Orbital G. | R | DM | 4 | -1 | 0 | 0 | 5 | 0 | -1 | 5 |
|  | Paracentral G. | L | SM | 7 | 2 | 3 | -1 | 0 | 3 | 3 | 4 |
|  | Postcentral G. | R | SM | 12 | -1 | 5 | 7 | 0 | 1 | 5 | 7 |
|  | Precentral G. | L | SM | 10 | 0 | 4 | 6 | 0 | 0 | 4 | 6 |
|  | Precentral G. | L | SM | 13 | 0 | 6 | 3 | 2 | 2 | 6 | 7 |
|  | Precentral G. | L | SM | 16 | 0 | 11 | 6 | -1 | 0 | 11 | 5 |
|  | Postcentral G. | L | SM | 13 | 0 | 2 | 8 | 0 | 3 | 2 | 11 |
|  | Postcentral G. | L | SM | 8 | -1 | 2 | 5 | 0 | 2 | 2 | 6 |
|  | Inferior Parietal Lobe | R | SM | 4 | 0 | -1 | 0 | 2 | 3 | -1 | 5 |
|  | Insula | R | CO/S | 14 | 1 | 6 | 3 | 2 | 2 | 2 | 12 |
|  | Superior Temporal G. | R | SM | 21 | 0 | 11 | 2 | 7 | 1 | 11 | 10 |
|  | Superior Temporal G. | R | SM | 6 | 1 | 2 | 0 | 3 | 0 | 2 | 4 |
|  | Postcentral G. | L | SM | 7 | 0 | 1 | 2 | 3 | 1 | 1 | 6 |
|  | Precentral G. | R | SM | 2 | -1 | 3 | 2 | -3 | 0 | -1 | 3 |
|  | Postcentral G. | R | SM | 10 | 0 | 7 | 1 | 0 | 2 | 7 | 3 |
|  | Postcentral G. | L | SM | 10 | 1 | 3 | 2 | 4 | 0 | 3 | 7 |
|  | Posterior Cingulate G. | L | DM | 5 | -1 | 0 | 1 | 4 | 1 | -1 | 6 |
|  | Cuneus | L | VIS | 15 | 2 | 11 | 1 | 0 | 1 | 1 | 14 |
|  | Precuneus | R | VIS | 15 | 1 | 11 | 1 | 1 | 1 | 1 | 14 |
|  | Middle Occipital | L | VIS | 7 | 2 | 2 | 0 | 1 | 2 | 0 | 7 |
| **Late Adolescence vs. Adulthood** | Parahippocampal G. | L | DM | 7 | -2 | 3 | 3 | 0 | 3 | -2 | 9 |
|  | Insula | R | SM | 1 | 0 | -3 | 1 | 3 | 0 | -3 | 4 |
|  | Middle Temporal G. | L | DM | 1 | -5 | 3 | 3 | -1 | 1 | -5 | 6 |
|  | Angular G | L | DM | 4 | -1 | 2 | 1 | 1 | 1 | -1 | 5 |
|  | Posterior Cingulate G. | L | DM | 3 | 1 | 3 | -1 | 0 | 0 | 1 | 2 |
|  | Superior Frontal G. | L | DM | 2 | -2 | 0 | 1 | 1 | 2 | -2 | 4 |
|  | Medial Frontal G. | R | DM | 8 | 4 | 1 | 1 | 2 | 1 | 4 | 4 |
|  | Medial Frontal G. | R | DM | 5 | 0 | 0 | 0 | 4 | 1 | 0 | 5 |
|  | Medial Frontal G. | L | DM | 4 | 1 | 1 | 0 | 2 | 0 | 1 | 3 |
|  | Medial Frontal G. | L | DM | 2 | 0 | -1 | 1 | 1 | 1 | 0 | 2 |
|  | Cuneus | L | VIS | 5 | 3 | 1 | -2 | 3 | 0 | -2 | 7 |
|  | Lingual G. | L | VIS | 8 | 5 | 2 | -3 | 2 | 2 | -3 | 11 |
|  | Lingual G. | R | VIS | 6 | 1 | 1 | 1 | 2 | 1 | 1 | 5 |
|  | Lingual G. | R | VIS | 3 | 1 | 0 | -1 | 3 | 0 | -1 | 4 |
|  | Inferior Parietal Lobe | L | FP | 1 | 2 | 1 | 0 | 4 | -6 | -6 | 7 |
|  | Medial Frontal G. | L | CO/S | 6 | 7 | 0 | 0 | -6 | 5 | -6 | 12 |
|  | Middle Frontal G. | L | FP | 3 | 0 | 4 | 0 | -1 | 0 | 0 | 3 |
